# Reliability of genomic predictions of complex human phenotypes

**DOI:** 10.1186/s12919-018-0138-5

**Published:** 2018-09-17

**Authors:** Arthur Porto, Juan M. Peralta, Nicholas B. Blackburn, John Blangero

**Affiliations:** 1 South Texas Diabetes and Obesity Institute, University of Texas Rio Grande Valley School of Medicine, One West University Blvd. Modular Building #100, Brownsville, TX 78250 USA; 2 Centre for Ecological and Evolutionary Synthesis, University of Oslo, Blindernveien 31, Oslo, Norway; 3 Menzies Institute for Medical Research, University of Tasmania, 17, Liverpool St., Hobart, TAS Australia

## Abstract

Genome-wide association studies have helped us identify a wealth of genetic variants associated with complex human phenotypes. Because most variants explain a small portion of the total phenotypic variation, however, marker-based studies remain limited in their ability to predict such phenotypes. Here, we show how modern statistical genetic techniques borrowed from animal breeding can be employed to increase the accuracy of genomic prediction of complex phenotypes and the power of genetic mapping studies.

Specifically, using the triglyceride data of the GAW20 data set, we apply genomic-best linear unbiased prediction (G-BLUP) methods to obtain empirical genetic values (EGVs) for each triglyceride phenotype and each individual. We then study 2 different factors that influence the prediction accuracy of G-BLUP for the analysis of human data: (a) the choice of kinship matrix, and (b) the overall level of relatedness. The resulting genetic values represent the total genetic component for the phenotype of interest and can be used to represent a trait without its environmental component.

Finally, using empirical data, we demonstrate how this method can be used to increase the power of genetic mapping studies. In sum, our results show that dense genome-wide data can be used in a wider scope than previously anticipated.

## Background

Genomic prediction (GP) refers to the use of genomic information for predicting an individual’s phenotype [1]. Several different approaches have been developed with the purpose of performing GP, such as marker-assisted selection (MAS) and genomic-best linear unbiased prediction methods (G-BLUP) [2]. MAS approaches have been widely successful when single genomic variants affect the trait of interest, but remain limited in their predictive capabilities for complex phenotypes [3]. Evidence suggests that complex traits are influenced by many genes, with effects that often fall below statistical significance thresholds [4]. As a consequence, the combined effects of variants identified through association only explains a small portion of the interindividual phenotypic differences [5]. G-BLUP–based methods, on the other hand, are not heavily influenced by statistical power, and have shown strong predictive power [6]. Traditionally, G-BLUP uses genomic relationships (ie, kinship) to estimate the empirical genetic value (EGV) of an individual. EGVs are increasingly being used in human genetic research, as they open the possibility of development of truly personalized medicine [7].

The generation of reliable EGV estimates constitutes one of their most important properties for the potential use of GP. Findings from the field of animal breeding strongly suggest that accuracy of GP can occasionally be low, and that the accuracy in relatedness estimates significantly affects the reliability of EGVs [8]. While pedigree kinship estimates have traditionally been the preferred measurement of relatedness, recent years have seen increased use of empirical kinships calculated from dense genome-wide data. Empirical kinships have the advantage of capturing distant relationships, preventing the exclusion of individuals with no genealogical record, and being less dependent on theoretical expectations [9]. The different kinship estimates, however, have not been properly compared using human data in terms of their reliability in the context of GP.

Here, using the distributed GAW20 data [10], we study 2 factors that affect the prediction accuracy of G-BLUP for the analysis of human data: (a) the choice of kinship matrix, and (b) the overall level of relatedness. We begin by describing the quality control methods used on the GAW data set. We then describe and compare 3 different kinship matrices in terms of the reliability of their EGV estimates, using analytical methods. We then assess whether overall levels of relatedness influence the accuracy of EGV estimates. With this analysis, we show that family data, together with the use of empirically derived kinship estimates, might increase the accuracy of GP of complex traits. Our results also show that G-BLUP methods might be used to increase the power of genetic linkage studies.

## Methods

### Initial processing

All of the analyses were done using the entirety of the distributed GAW20 dataset. The initial GAW20 phenotype data file (1102 individuals) presented 4 triglyceride (TG) measurements (trr1 to trr4), representing TG levels at 4 different time points, 2 pre- and 2 post-fenofibrate intervention. To reduce the effects of measurement error, pre- and posttreatment TG replicates were averaged. The resulting file was, together with the pedigree file, converted to SOLAR (Sequential Oligogenic Linkage Analysis Routines) format [11].

The physical coordinates for GAW20 genotypes were converted to release 19 of the human genome (hg19) from UCSC. PREST-Plus [12] was then used to identify erroneous samples recorded in the pedigree relationships. This curated data set was posteriorly converted to input formats for 2 widely used software packages, LDAK [13] and IBDLD [9].

### Pedigree and empirical kinships

Pedigree kinship estimates were obtained from the original pedigree data file using SOLAR. Two different empirical kinship matrices were then calculated from the curated genotype data using LDAK version 4.9 and IBDLD version 3.33. Both software packages attempt to account for the linkage disequilibrium (LD) present in dense genotype data. However, they differ in how they account for LD. IBDLD uses a hidden Markov model to estimate identity-by-descent probabilities conditional on multilocus genotype information. LDAK, on the other hand, assesses local patterns of LD prior to kinship estimation, and then uses that information to give each single-nucleotide polymorphism (SNP) a specific weight during kinship calculations that accounts for the extent to which the genetic signal is replicated by its neighboring SNPs. The empirical kinship estimates from LDAK and IBDLD were both weighted and scaled, ensuring the diagonal elements were equal to 1.

### G-BLUPs

The pre- and posttreatment TG levels, and relevant covariates (age, sex, study center, and smoking) were exported to TASSEL [14] together will all 3 kinship matrices. G-BLUPs based on each of the 3 matrices were then calculated using the *Genomic Selection* function. G-BLUPs are calculated by solving the mixed model equation:

$$ \mathrm{y}=\mathrm{Xb}+\mathrm{Zu}+\mathrm{e} $$

where, *y* is a vector of phenotypic observations; *b* is a vector of fixed effects with design matrix *X*; *u* is a vector of random polygenic effects (EGV) with design matrix *Z*; and *e* is a vector of residual effects. There are 2 important features of G-BLUP worth noting. First, the variance structure of *u* is proportional to the relationship matrix and, therefore, we should expect *u* to be directly affected by kinship estimates. Second, G-BLUP does not directly make assumptions regarding the number of loci underlying the traits of interest. However, G-BLUP does assume that the underlying loci have similar effect sizes, which is not an accurate assumption when the number of underlying loci is small. Given the polygenic nature of TG phenotypes, however, we do not expect this assumption to have been violated in our case.

A single common criterion was used to assess each matrix’s performance in producing accurate EGV estimates. In particular, we estimated the accuracy of individuals’ EGV based on the prediction error variance (PEV). In the absence of statistical bias, PEV is equal to the mean squared error (MSE). The accuracy was estimated as $\mathrm{EA}=\sqrt{1- PEV/\sigma 2\mathit{\mathsf{g}}}$, where *σ2g* is the additive genetic variance of the base population [1]. This accuracy measure can be interpreted as reflecting the extent to which individuals’ EGVs may change when more detailed information about them becomes available, such as the addition of a close relative to the analyses. A small prediction error variance indicates that additional information would not lead to a change in the EGV estimate and, therefore, that the estimate is reliable.

### Second-degree relatives’ approximation

Research in animal breeding suggests that the number of relatives in a pedigree can influence the accuracy of estimated EGVs [8]. To test that hypotheses, we regressed EA estimates on the number of second-degree relatives (SDRs). SDR was calculated here, for each individual, as the approximate number of second-degree relatives an individual has on the total data set. This approximation was obtained by counting the number of pairwise kinship coefficients between an individual and the rest of the population that are higher than 0.25.

### Linkage mapping

The decomposition of a trait into its genetic and environmental components opens up the possibility of increasing the genetic signal in linkage studies through the removal of structured environmental effects. Therefore, we here regressed the linkage signals obtained by using EGVs as traits on the results obtained from traditional genetic mapping (see Peralta et al. [15] for details of the mapping procedures). Regression slopes significantly higher than 1 were interpreted as indicating increased power for detecting genetic linkage.

## Results

### Initial processing

PREST-Plus identified a total of 6 potentially erroneous samples when taking into account the relationships within pedigrees. To prevent these erroneous samples from influencing the downstream analyses, they were removed from the original GAW20 data set (5604, 8117, 1927, 4078, 3621, 8117). See Blackburn et al. [16] for details.

### Accuracy of TG EGVs pre- and posttreatment

**Fig. 1:**
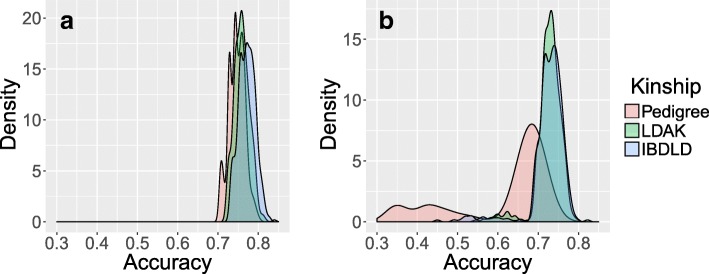
Reliability in EGV estimates using both (**a**) pre- and (**b**) posttreatment TG levels, when comparing across the different kinship matrices (Pedigree, LDAK, IBDLD). Distributions are illustrated using kernel density estimates (KDEs), as implemented in the *ggplot2* R package

Accuracy estimates (Fig. 1) suggest that both pre- and posttreatment TG levels can be fairly accurately predicted based on their kinship matrices, regardless of whether IBDLD, LDAK, or pedigree kinship was used. Accuracy ranged from 0.3 to 0.84, depending on the individual. Overall, the pedigree kinship matrix resulted in slightly lower average accuracy than the remaining matrices. Likewise, posttreatment TG are less-reliably estimated than pretreatment TG, largely because of individuals with missing phenotypes (lower tail values). Finally, the IBDLD-based kinship estimates are closer to LDAK than to pedigree estimates.

### Association between EGV reliability and the number of relatives in the pedigree

**Fig. 2:**
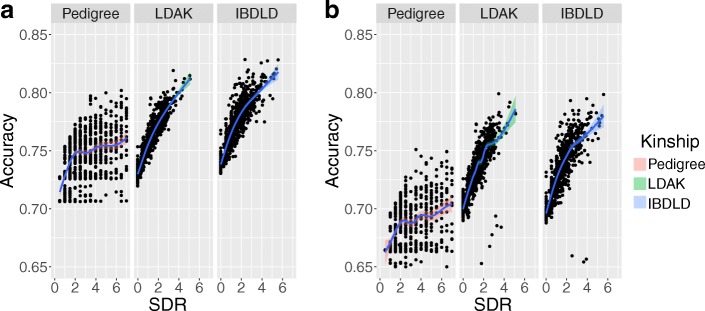
Local regressions of the accuracy in individual estimates of EGVs on the number of SDRs, when using both (**a**) pre- and (**b**) posttreatment TG levels, as well as different kinship matrices (Pedigree, LDAK, IBDLD) Note the closer fit of empirically derived EGVs when compared to pedigree-based EGVs

When regressing accuracy estimates on the number of SDRs (Fig. 2), we found a close relationship between the accuracy of EGV estimates and the number of relatives an individual possesses in the pedigree. The overall fit of linear regressions is higher among empirically derived EGVs (~ 75% for pre-TG) than pedigree-based EGVs (~ 20% for pre-TG). Outliers in the posttreatment TG data are associated with individuals with missing phenotypes.

### Linkage mapping

**Fig. 3:**
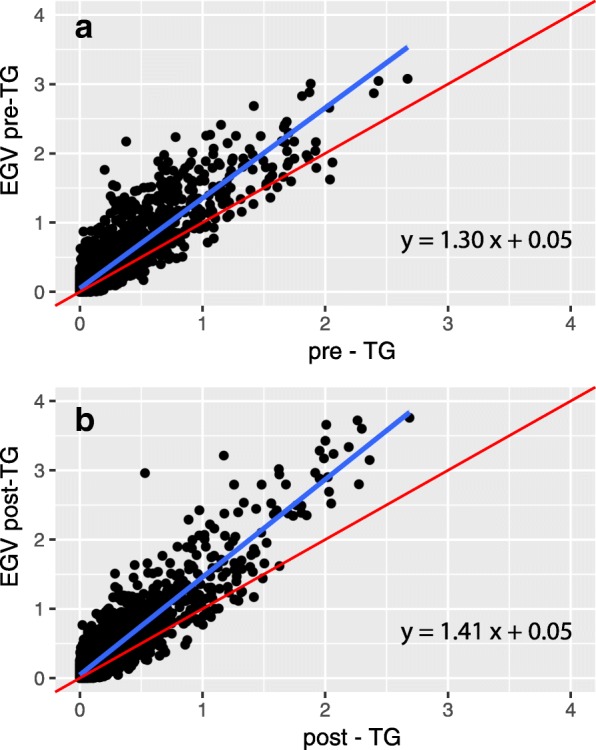
Linear regressions of (**a**) pre- and (**b**) post-TG LOD scores obtained from EGV-based linkage scans on the traditional linkage scan LODs. Red line indicates the 1:1 line and the blue line indicates the best-fitting regression line. Regression equations are shown in the bottom right corner

When regressing logarithm of odds (LOD) scores obtained from EGV-based linkage scans on the traditional linkage scan LODs (pre-TG Fig. 3a; post-TG Fig. 3b), we found a close relationship between the LOD scores across the 2 different approaches. The overall fit of the linear regression indicates that no substantial change in locus rank occurred when using EGVs as traits in the linkage scans. However, the slope of the regression is significantly higher than 1 for both pre- (*p* < 0.001) and posttreatment- TG levels (*p* < 0.001), indicating that the use of EGVs considerably enhanced the genetic signal in both cases.

## Discussion

Reliable prediction of complex human phenotypes or diseases will be essential to attain the objective of truly personalized medicine. Marker-informed prediction based on SNPs has already become commonplace [3, 17–19] and increasingly sophisticated. However, most of the studies using small SNP sets have generally explained very little of the total variation in complex traits, with values often much lower than 4% of the total phenotypic variation.

Genome-wide approaches, on the other hand, are still rare, but have already helped us attain much higher predictive power. Yang et al. [20], for example, used a total of 294,831 SNPs scored in approximately 4000 individuals to show that such a SNP set could explain as much as 45% of the total variance.

In this study, we aimed to show how statistical techniques borrowed from animal breeding could be employed to predict complex phenotypes with relatively high accuracy (see Fig. 1). Furthermore, we tested the overall effect of choice of kinship matrix and pedigree relatedness in influencing the accuracy of G-BLUP. Although significant, the increase in accuracy obtained by using empirically derived kinships is likely not substantial enough to solely justify the price of scoring dense marker data. However, when dense marker data are available, the results presented here suggest empirically derived kinship matrices can be useful in increasing the accuracy of EGV estimates. This increase is particularly evident in individuals with missing phenotypes, who form the lower tail of the distribution of accuracies (see Fig. 1b). Empirical kinships can capture distant relatedness and, therefore, improve the accuracy of the phenotypic prediction of individuals with missing phenotype by using their relative’s information.

In general, prediction accuracy increases with the number of relatives an individual possesses in the data set (see Fig. 2). This is particularly true for empirically derived kinship estimates, as genomic data allows one to capture more distant or nuanced relationships between individuals. Individuals with missing phenotypes, however, have more poorly estimated EGV values, regardless of choice of kinship matrix.

In any case, our results also suggest that using EGVs in linkage studies might be a fruitful way to increase the power to detect genomic regions underlying complex traits. By removing the structured environmental variance from the phenotypic variance, we see a pronounced increased in LOD scores of the linkage model. It should be noted, however, that a proper test of this hypothesis is to start with a system in which the genomic variants are known a priori.

Because the GAW20 data set is composed of mostly unrelated individuals, one could anticipate that GP based on more closely related individuals would generate highly accurate EGV estimates. In other words, family-based studies might represent a particularly useful starting point when attempting to use EGVs in the analyses of complex traits or in personalized medicine.

## Conclusions

Our analysis of the GAW20 data set shows that dense genome-wide SNP data can be used to accurately estimate EGVs for use in personalized medicine or to increase the power of linkage scans. EGVs estimated based on empirical kinship matrices are slightly more reliable than pedigree-based matrices, largely as a consequence of their ability to capture distant relationships among individuals. Similarly, the prediction accuracy increases with the number of relatives an individual possesses in the data set. In sum, family-based studies, with empirically derived kinships, might be the ideal study design for the application of GP of complex traits in human health research.

## References

[1] Gorjanc G, Bijma P, Hickey JM (2015). Reliability of pedigree-based and genomic evaluations in selected populations. Genet Sel Evol.

[2] Xu S, Zhu D, Zhang Q (2014). Predicting hybrid performance in rice using genomic best linear unbiased prediction. Proc Natl Acad Sci U S A.

[3] Van Hoek M, Dehghan A, Witteman JC, Van Duijn CM, Uitterlinden AG, Oostra BA, Hofman A, Sijbrands EJ, Janssens AC (2008). Predicting type 2 diabetes based on polymorphisms from genome-wide association studies. Diabetes.

[4] McCarthy MI, Abecasis GR, Cardon LR, Goldstein DB, Little J, Ioannidis JP, Hirschhorn JN (2008). Genome-wide association studies for complex traits: consensus, uncertainty and challenges. Nat Rev Genet.

[5] Zhao J, Bradfield JP, Li M, Wang K, Zhang H, Kim CE, Annaiah K, Glessner JT, Thomas K, Garris M (2009). The role of obesity-associated loci identified in genome-wide association studies in the determination of pediatric BMI. Obesity (Silver Spring).

[6] Daetwyler HD, Swan AA, van der Werf JH, Hayes BJ (2012). Accuracy of pedigree and genomic predictions of carcass and novel meat quality traits in multi-breed sheep data assessed by cross-validation. Genet Sel Evol.

[7] Lee SH, Weerasinghe WM, Wray NR, Goddard ME, van der Werf JH (2017). A better design for stratified medicine based on genomic prediction. Sci Rep.

[8] Clark SA, Hickey JM, Daetwyler HD, van der Werf JH (2012). The importance of information on relatives for the prediction of genomic breeding values and the implications for the makeup of reference data sets in livestock breeding schemes. Genet Sel Evol.

[9] Han L, Abney M (2011). Identity by descent estimation with dense genome-wide genotype data. Genet Epidemiol.

[10] Irvin MR, Zhi D, Joehanes R, Mendelson M, Aslibekyan S, Claas SA, Thibeault KS, Patel N, Day K, Jones LW (2014). Epigenome-wide association study of fasting blood lipids in the genetics of lipid lowering drugs and diet network study. Circulation.

[11] Almasy L, Blangero J (1998). Multipoint quantitative-trait linkage analysis in general pedigrees. Am J Hum Genet.

[12] Sun L, Dimitromanolakis A (2014). PREST-plus identifies pedigree errors and cryptic relatedness in the GAW18 sample using genome-wide SNP data. BMC Proc.

[13] Speed D, Cai N, UCLEB Consortium JMR, Nejentsev S, Balding DJ (2017). Re-evaluation of SNP heritability in complex human traits. Nat Genet.

[14] Bradbury PJ, Zhang Z, Kroon DE, Casstevens TM, Ramdoss Y, Buckler ES (2007). TASSEL: software for association mapping of complex traits in diverse samples. Bioinformatics.

[15] Peralta JM, Blackburn NB, Porto A, Blangero J, Charlesworth J. Genome**-**wide linkage scan for loci influencing plasma triglyceride levels. BMC Proc. 2018;12(Suppl 9). 10.1186/s12919-018-0137-6. 10.1186/s12919-018-0137-6 PMC6157192 30275898

[16] Blackburn NB, Porto A, Peralta JM, Blangero J. Heritability and genetic associations of triglyceride and HDL-C levels using pedigree-based and empirical kinships. BMC Proc. 2018;12(Suppl 9). 10.1186/s12919-018-0133-x. 10.1186/s12919-018-0133-x PMC6157025 30263045

[17] Seshadri S, Fitzpatrick AL, Ikram MA, DeStefano AL, Gudnason V, Boada M, Bis JC, Smith AV, Carrasquillo MM, Lambert JC (2010). Genome-wide analysis of genetic loci associated with Alzheimer disease. JAMA.

[18] Valenzuela RK, Henderson MS, Walsh MH, Garrison NA, Kelch JT, Cohen-Barak O, Erickson DT, John Meaney F, Bruce Walsh J, Cheng KC (2010). Predicting phenotype from genotype: normal pigmentation. J Forensic Sci.

[19] Willer CJ, Speliotes EK, Loos RJ, Li S, Lindgren CM, Heid IM, Berndt SI, Elliott AL, Jackson AU, Lamina C (2009). Six new loci associated with body mass index highlight a neuronal influence on body weight regulation. Nat Genet.

[20] Yang J, Benyamin B, McEvoy BP, Gordon S, Henders AK, Nyholt DR, Madden PA, Heath AC, Martin NG, Montgomery GW (2010). Common SNPs explain a large proportion of the heritability for human height. Nat Genet.

